# The Effect of Dual-Hemisphere Transcranial Direct Current Stimulation Over the Parietal Operculum on Tactile Orientation Discrimination

**DOI:** 10.3389/fnbeh.2017.00173

**Published:** 2017-09-20

**Authors:** Shuhei Fujimoto, Satoshi Tanaka, Ilkka Laakso, Tomofumi Yamaguchi, Noriko Kon, Takeo Nakayama, Kunitsugu Kondo, Ryo Kitada

**Affiliations:** ^1^Tokyo Bay Rehabilitation Hospital Chiba, Japan; ^2^Department of Public Health, Kyoto University Graduate School of Medicine Kyoto, Japan; ^3^Link & Communication Inc. Tokyo, Japan; ^4^Laboratory of Psychology, Hamamatsu University School of Medicine Shizuoka, Japan; ^5^Department of Electrical Engineering and Automation, Aalto University Espoo, Finland; ^6^Department of Rehabilitation Medicine, Keio University School of Medicine Tokyo, Japan; ^7^Department of Neuroscience and Pharmacology, University of Copenhagen Copenhagen, Denmark; ^8^Department of Physical Therapy, Yamagata Prefectural University of Health Sciences Yamagata, Japan; ^9^Department of Therapy, Kawakita Rehabilitation Hospital Tokyo, Japan; ^10^Division of Psychology, School of Social Sciences (SSS), College of Humanities, Arts, & Social Sciences, Nanyang Technological University Singapore, Singapore

**Keywords:** cortical plasticity, inter-hemispheric inhibition (IHI), somatosensory cortex, tactile, transcranial direct current stimulation (tDCS), transcranial magnetic stimulation (TMS)

## Abstract

The parietal operculum (PO) often shows ipsilateral activation during tactile object perception in neuroimaging experiments. However, the relative contribution of the PO to tactile judgment remains unclear. Here, we examined the effect of transcranial direct current stimulation (tDCS) over bilateral PO to test the relative contributions of the ipsilateral PO to tactile object processing. Ten healthy adults participated in this study, which had a double-blind, sham-controlled, cross-over design. Participants discriminated grating orientation during three tDCS and sham conditions. In the dual-hemisphere tDCS conditions, anodal and cathodal electrodes were placed over the left and right PO. In the uni-hemisphere tDCS condition, anodal and cathodal electrodes were applied over the left PO and contralateral orbit, respectively. In the tDCS and sham conditions, we applied 2 mA for 15 min and for 15 s, respectively. Computational models of electric fields (EFs) during tDCS indicated that the strongest electric fields were located in regions in and around the PO. Compared with the sham condition, dual-hemisphere tDCS improved the discrimination threshold of the index finger contralateral to the anodal electrode. Importantly, dual-hemisphere tDCS with the anodal electrode over the left PO yielded a decreased threshold in the right finger compared with the uni-hemisphere tDCS condition. These results suggest that the ipsilateral PO inhibits tactile processing of grating orientation, indicating interhemispheric inhibition (IHI) of the PO.

## Introduction

Humans are remarkably capable of recognizing objects by touch (Klatzky et al., 1985). This is achieved by extracting object properties regarding the texture, shape and orientation of an object. Previous neuroimaging studies have characterized the brain networks underlying tactile object processing (Kitada, 2016). However, as most of these studies relied on correlational measures, the functional relevance of these regions is not well understood.

One unresolved issue is the relationship between the bilateral somatosensory cortices during tactile object perception. For instance, neurons with receptive fields for bilateral body parts are present in the secondary somatosensory cortex (S2) and the caudal part of the primary somatosensory cortex (S1; area 2) in non-human primates (Iwamura et al., 1994; Taoka et al., 2016). Previous neuroimaging studies have shown that tactile stimulation of the right hand causes bilateral activation of the parietal operculum (PO), including the S2 (Roland et al., 1998; Karhu and Tesche, 1999; Burton et al., 2004; Kitada et al., 2005; Yang et al., 2017). This activation can be interpreted to indicate that the bilateral somatosensory cortices work in harmony to perform tasks involving only one hand.

However, it has been well established that each hemisphere of the primary motor cortex (M1) inhibits activity in the other hemisphere (Allison et al., 2000; Fregni et al., 2005; Boggio et al., 2008; Hayashi et al., 2008; Kobayashi et al., 2009; Ni et al., 2009). Such interhemispheric inhibition (IHI) is considered critical for the suppression of unwanted motor activity in opposite limbs during unilateral movements and learning (Duque et al., 2005). This inhibition may also occur in the somatosensory cortex, to inhibit sensory processing of the irrelevant side of the body for efficient processing of sensory inputs on the relevant side. Previous studies have examined this issue in the S1 (Seyal et al., 1995; Clarey et al., 1996; Blankenburg et al., 2008; Ragert et al., 2011; Brodie et al., 2014; Fujimoto et al., 2014). In contrast, few studies have investigated whether the two hemispheres of the PO work in competition or in harmony.

One way to address this issue is the use of transcranial direct current stimulation (tDCS) techniques. tDCS is a non-invasive technique that stimulates brain areas via the application of weak direct currents through the scalp (Priori et al., 1998; Nitsche and Paulus, 2000). tDCS can increase or decrease the excitability of a stimulated cortical region depending on the polarity of stimulation (Nitsche and Paulus, 2000). The immediate effects of tDCS are thought to depend on subthreshold resting membrane potential changes, whereas the aftereffects of tDCS are thought to be caused by shifts in intracortical inhibition and facilitation (Nitsche et al., 2005). Previous studies have shown that tDCS can modulate somatosensory evoked potentials (SEPs) and tactile performance in healthy volunteers (Matsunaga et al., 2004; Rogalewski et al., 2004; Dieckhofer et al., 2006; Ragert et al., 2008). For instance, cathodal tDCS over the S1 decreased SEP amplitude (Dieckhofer et al., 2006), while anodal tDCS over M1 increased SEP amplitude (Matsunaga et al., 2004). Behaviorally, cathodal tDCS over S1 decreased participant performance in a task involving tactile discrimination of vibration frequency (Rogalewski et al., 2004), while anodal tDCS over S1 improved tactile orientation discrimination (Ragert et al., 2008). Unlike post-stroke motor recovery (Kang et al., 2016; Lefebvre and Liew, 2017), few studies have investigated the effect of tDCS on somatosensory recovery after stroke (Fujimoto et al., 2016).

We previously examined the interhemispheric relationship between somatosensory cortices using dual-hemisphere tDCS. In this protocol, both hemispheres were simultaneously stimulated to excite one hemisphere via anodal tDCS while inhibiting the other with cathodal tDCS (Vines et al., 2008). If activation in both hemispheres plays a similar role in tactile object perception, then dual-hemisphere tDCS would be expected to elicit reduced performance compared with conventional single-hemisphere tDCS. Alternatively, if the two hemispheres inhibit one another, dual-hemisphere tDCS would be expected to elicit increased task performance compared with single-hemisphere tDCS. We recently found that dual-hemisphere tDCS of the S1 in healthy participants elicited greater performance in a tactile discrimination task compared with uni-hemisphere tDCS, supporting the involvement of IHI (Fujimoto et al., 2014). However, to the best of our knowledge, no previous study has examined the effect of bilateral vs. uni-hemisphere tDCS in the PO.

The goal of the present study was to examine the effects of dual-hemisphere tDCS over the PO on tactile object processing, compared with the effects of uni-hemisphere tDCS over the same region in healthy volunteers. We adopted the grating orientation task (GOT), which enables direct comparisons with our previous studies (Fujimoto et al., 2014, 2016). We hypothesized that if the two hemispheres of the PO work in harmony, dual-hemispheric tDCS would decrease task performance (i.e., increase the GOT threshold) compared with uni-hemispheric tDCS and sham control stimulation. Alternatively, if the two hemispheres function in competition, dual-hemispheric tDCS should elicit increased task performance (lower GOT threshold) compared with uni-hemispheric tDCS stimulation and sham controls. Finally, we estimated the electric fields (EFs) in the brain during tDCS by constructing computational models (Laakso et al., 2016).

## Materials and Methods

### Participants

Ten healthy naïve volunteers (seven males and three females; mean age ± standard deviation (SD) = 24.9 ± 1.3 years) participated in the study. The number of participants was determined by statistical power analysis (GPower 3.1, Faul et al., 2007). The effect size was estimated according to the two previous studies that demonstrated the effects of dual-hemisphere tDCS over the PO of stroke patients (Fujimoto et al., 2016) and over the S1 of healthy participants (mean *d*_z_ = 1.43). All participants were right hand dominant according to the Edinburgh Handedness Inventory (Oldfield, 1971), and none had a history of psychiatric or neurological illness. This study was carried out in accordance with the recommendations of the local ethics committee of Tokyo Bay Rehabilitation Hospital. All participants gave written informed consent in accordance with the Declaration of Helsinki. The protocol was approved by the local ethics committee of Tokyo Bay Rehabilitation Hospital.

### Transcranial Direct Current Stimulation (tDCS)

We delivered direct current tDCS using a DC Stimulator Plus (NeuroConn, Germany) with two sponge surface electrodes (each with a surface area of 25 cm^2^). The stimulation intensity was 2 mA based on our previous study (Fujimoto et al., 2016). Two milliampere stimulation has been reported to minimize the intra- and inter-individual variability of the tDCS effect (Wiethoff et al., 2014; Laakso et al., 2015) and to induce consistent intra- and inter-individual increases of M1 excitability (Ammann et al., 2017). In all conditions except for the sham condition, we applied a direct current for 15 min. During stimulation, we gradually increased the current from 0 mA to 2 mA for the first 15 s and gradually decreased the current from 2 mA to 0 mA for the last 15 s. The current density at the stimulation electrodes was 0.08 mA/cm^2^. These parameters are in accordance with a safety criterion and are far below the threshold for tissue damage (Nitsche et al., 2003; Poreisz et al., 2007). For the sham condition, we used the same procedure but applied current for only 15 s (Gandiga et al., 2006).

To identify the PO, we obtained T1 anatomical images for all participants using magnetic resonance imaging (Intera 1.5 T, Philips, Netherlands) prior to the tDCS experiment. For each participant, the centers of the stimulation electrodes were placed over the PO, identified via the individual T1 anatomical images. The target area was localized using a frameless stereotaxic navigation system (Brainsight2, Rogue Research Inc., Montreal, Canada). Our previous study confirmed that this procedure affected the SEP of the PO, but not the hand area of S1 (Nakagawa et al., 2017).

### Experimental Procedure

We adopted a double-blind, crossover, sham-controlled experimental design (Hummel et al., 2005; Gandiga et al., 2006). The experiment involved four types of interventions: two types of dual hemisphere tDCS, uni-hemisphere tDCS and sham stimulation. In one type of dual tDCS, the anode and cathode electrodes were placed over the left and right PO, respectively (Dual-Anodal-Left condition). In the other type of dual tDCS, these electrodes were placed over the opposite hemispheres (Dual-Anodal-Right condition). In uni-hemisphere tDCS, we administered anodal tDCS over the left PO and cathodal tDCS over the forehead above the contralateral orbit (Uni-Anodal-Left condition). This uni-hemisphere stimulation was used for comparison with Dual-Anodal-Left condition to examine the contribution of the ipsilateral PO when the right finger was stimulated. Each participant underwent four sessions, each of which involved one type of intervention and was performed between 9:00–18:00 on the same day. The tDCS sessions were separated by at least 3 days and the order of the sessions was counterbalanced among participants. In all sessions, we placed three electrodes in the same positions (two electrodes over the PO and the one electrode on the orbit) on all participants. The experimenter who applied the tDCS then discreetly connected the two active electrodes to the DC Stimulator. Thus, neither the experimenter who measured task performance nor the participant knew which stimulation type was applied during each trial.

### Grating Orientation Task (GOT)

To examine the effect of tDCS over the PO, we used the GOT (Van Boven and Johnson, 1994). The GOT is widely used as a measure of tactile spatial acuity (Sathian et al., 1997; Goldreich and Kanics, 2003; Ragert et al., 2008). During this task, participants sat blindfolded on a chair in a comfortable position. The tactile stimuli were five hemispherical plastic domes with grooves of different widths (0.5, 0.75, 1.0, 1.2 and 1.5 mm) cut into their surfaces (Tactile Acuity Grating, Miyuki Giken). We used a custom-made device to control the up-down movements of the domes, such that one dome was presented to the participant in each trial. A single skilled investigator tested all participants to minimize possible variance of stimulation.

Each session involved three task blocks, each of which was presented before the tDCS (Pre), during the tDCS (During), and 10 min after the tDCS (Post 10 min). Each task block in the dual-hemisphere tDCS and sham conditions consisted of 200 trials: 100 trials each for the left and right index fingers. The participants completed all trials for one finger first, followed by all trials with the other finger. The order of the fingers was counterbalanced. Each task block of the uni-hemisphere tDCS contained 100 trials for the right finger. For each finger, one of the five domes was successively presented during 20 trials. We presented the domes in the following order: 1.5 mm, 1.2 mm, 1.0 mm, 0.75 mm and 0.5 mm. Each dome was presented in each of two orientations 10 times and the order of the orientations was pseudo-randomized (20 trials for two orientations × 5 domes = 100 trials for each finger). In each trial, the groove of the dome was randomly oriented in one of two directions: orthogonal or parallel to the axis of the index finger. The dome was applied with moderate force onto the fingertip for 2 s. After the dome left the finger, participants were asked to verbally report whether the orientation of the grating of the presented dome was parallel or orthogonal in a two-alternative forced choice paradigm. Before the first session of the experiment, the participants were familiarized with the task.

### Post-Experiment Questionnaire

To assess the participants’ subjective state during tDCS, we asked them to complete a questionnaire that measured their levels of attention, fatigue, pain, sleepiness and discomfort at the end of each session. The questionnaire had a four-point scale (e.g., attention [1 = no distraction; 4 = highest level of distraction]).

### Analysis

We calculated the groove width for which 75% of the responses were correct in each block. We defined this width as the discrimination threshold and used it as a primary outcome measurement. We calculated the threshold using a linear interpolation method, as follows (Ragert et al., 2008).

$${\text{Threshold = G}}_{\text{below}}\text{ + }\frac{\left(0.75\;-\;{\text{P}}_{\text{below}})\;\times \;({\text{G}}_{\text{above}}\;-\;{\text{G}}_{\text{below}}\right)}{{\text{(P}}_{\text{above}}\;-\;{\text{P}}_{\text{below}}\text{)}}$$

G_below_: the highest grating spacing for which the participant responded correctly less than 75% of the trials.
G_above_: the lowest grating spacing for which the participant responded correctly more than 75% of the trials.
P_below_: the percentage of correct responses for G_below_.
P_above_: the percentage of correct responses for G_above_.

We then statistically evaluated the patterns of the thresholds using SPSS software (version 18; IBM Corporation, Armonk, NY, USA). We conducted two analyses. The purpose of the first analysis was to examine whether dual-hemisphere tDCS over the PO affected thresholds of healthy participants in a similar way to the stroke patients in our previous study (Fujimoto et al., 2016). In this analysis, we compared the effects of dual hemisphere tDCS with that of the sham condition. In the second analysis, we tested the main hypothesis, that dual-hemisphere tDCS would produce a stronger effect than uni-hemisphere tDCS. In both analyses, we initially conducted an analysis of variance (ANOVA), then conducted *post hoc* pairwise comparisons using Dunnett’s correction (two-tailed).

### Electric Field Modeling

We estimated the EFs that were produced by dual- and single-hemisphere electrodes in the brain, as in our previous study (Laakso et al., 2016). First, the cortical EFs were numerically calculated in 62 individual magnetic-resonance-imaging (MRI)-based anatomical models using the finite-element method. The sizes and locations of the electrodes were similar to those in the actual experiment (Figure 3A). Other parameters of the computer simulations were identical to our previous study (Laakso et al., 2016). The individual EFs were then registered with each other and mapped to the standard brain template (Fonov et al., 2009, 2011) using FreeSurfer image analysis software (Dale et al., 1999; Fischl et al., 1999; Fischl and Dale, 2000) and the spherical demons algorithm (Yeo et al., 2010). This allowed us to determine the population-average EFs and their variability in standard brain space.

**Figure 1:**
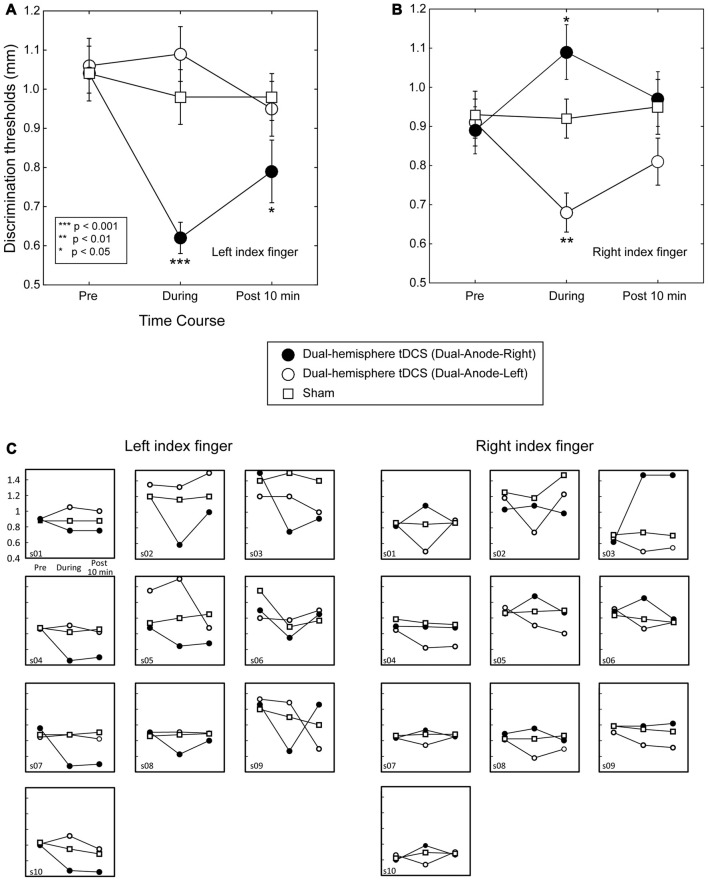
Grating orientation discrimination in dual-hemisphere vs. sham transcranial direct current stimulation (tDCS). The mean grating orientation threshold for each intervention is plotted as a time course with bars indicating standard error of the mean (SEM). Asterisks indicate significant differences in the threshold relative to sham control. **(A)** Compared with sham tDCS, Dual-Anode-Right tDCS (black circle) significantly improved the grating orientation threshold of the left finger during (During) and 10 min after the stimulation (Post 10 min). **(B)** Compared with sham tDCS, Dual-Anodal-Left tDCS (white circle) significantly improved the grating orientation threshold of the right finger during the stimulation. Conversely, Dual-Anodal-Right tDCS (black circle) significantly increased the grating orientation threshold during the stimulation. **(C)** Results of each individual participant.

**Figure 2:**
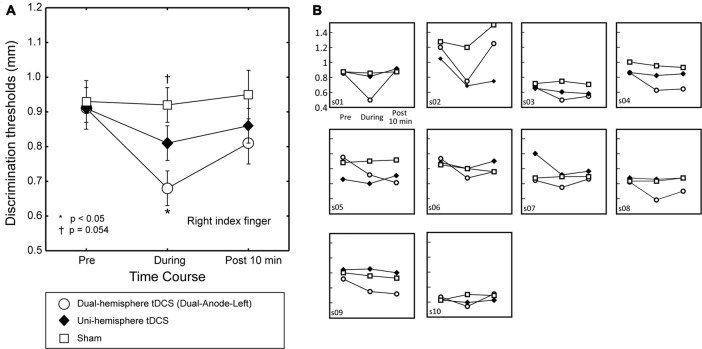
The effect of dual-hemisphere vs. uni-hemisphere tDCS on grating orientation threshold. The mean grating orientation threshold of the right finger in each intervention is plotted as a time course with bars indicating the SEM. **(A)** Asterisks indicate significant differences in the threshold relative to uni-hemisphere tDCS. Compared with uni-hemisphere tDCS, Dual-Anode-Left tDCS (white circle) elicited a significantly lower threshold during the stimulation (During). There was a trend towards a significant difference between the uni-hemisphere tDCS and sham conditions during the stimulation. **(B)** Results of each individual participant.

**Figure 3:**
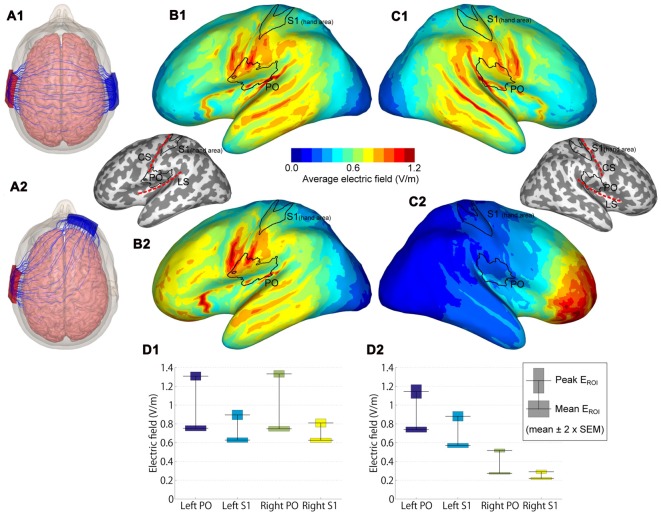
Modeled electric fields (EFs) of dual-hemisphere and uni-hemisphere tDCS. **(A)** Streamlines show the direction of the electric current in an example subject for dual-hemisphere **(A1)** and uni-hemisphere **(A2)** electrode montages. **(B)** Group-average EFs over 62 anatomical models of the left hemisphere.** (B1,B2)** indicate the EFs produced by dual-hemisphere and uni-hemisphere tDCS, respectively. Inset shows the locations of the regions of interest (ROIs) primary somatosensory cortex (S1 hand area and parietal operculum (PO)) relative to the gyrification pattern and central (CS) and lateral (LS) sulci. **(C)** Group-average EFs in the right hemisphere (**C1** for dual-hemisphere tDCS and **C2** for uni-hemisphere tDCS).** (D)** EFs in each ROI (**D1** for dual-hemisphere tDCS and **D2** for uni-hemisphere tDCS). The horizontal lines show the group-mean values of the peak and mean EF. Bars indicate ±2 × SEM.

To compare the EFs in the PO and S1, regions of interest (ROIs) were defined as follows. We initially defined S1 and PO from the SPM anatomy toolbox (Eickhoff et al., 2005). We then limited the S1 to the hand area, in which Montreal Neurological Institute (MNI) Z coordinates ranged from *Z* = 45 to *Z* = 65. This range of Z coordinates was determined according to anatomical landmarks (i.e., inverse omega shape on horizontal sections) and previous findings (Kitada et al., 2005, 2006; Yang et al., 2017). In each ROI, we reported the peak and mean EFs, calculated as the absolute value of the EF. The peak EF was defined as the EF at the point with the maximum average EF, and the mean EF was defined as the EF averaged over each ROI. Paired *t*-tests (two-tailed) with Bonferroni-correction were used for statistical testing.

## Results

### The Effects of Dual-Hemisphere tDCS Compared with the Sham Condition

Figure 1 left shows the effect of dual-hemisphere tDCS over the PO compared with that of the sham stimulation. The obtained data are available in Supplementary Tables S1, S2.

We performed a three-way repeated measures ANOVA on grating thresholds, with the factors of Finger (right and left finger), Intervention (Dual-Anodal-Right, Dual-Anodal-Left, and sham) and Time (Pre, During and Post 10 min). This analysis revealed a significant three-way interaction (*F*_(4,36)_ = 18.42, *P* < 0.001, $\eta_{\text{p}}^{2}$ = 0.67); significant two-way interactions of Finger *×* Intervention (*F*_(2,18)_ = 19.84, *P* < 0.001, $\eta_{\text{p}}^{2}$ = 0.69) and of Finger *×* Time (*F*_(2,18)_ = 6.32, *P* = 0.008, $\eta_{\text{p}}^{2}$ = 0.41); and a significant main effect of Time (*F*_(2,18)_ = 6.19, *P* = 0.009, $\eta_{\text{p}}^{2}$ = 0.41). As we found a significant three-way interaction, we examined the effects of tDCS for each finger separately.

#### The Effect of tDCS on the Left Finger

A two-way repeated-measures ANOVA (three levels of Intervention *×* three levels of Time) revealed significant main effects of Intervention (*F*_(2,18)_ = 13.80, *P* < 0.001, $\eta_{\text{p}}^{2}$ = 0.61) and Time (*F*_(2,18)_ = 12.57, *P* < 0.001, $\eta_{\text{p}}^{2}$ = 0.58). However, this analysis also revealed a significant two-way interaction (*F*_(4,36)_ = 8.90, *P* < 0.001, $\eta_{\text{p}}^{2}$ = 0.50). We conducted *post hoc* pairwise comparisons with Dunnett’s correction (two-tailed) to compare each tDCS effect with the sham condition. This analysis revealed no significant differences between the interventions before tDCS was applied (*P-values > 0.9)*. In contrast, the Dual-Anode-Right condition had a significantly lower grating threshold than the sham condition during tDCS (*P* < 0.001, *d*_z_ = 1.92) and 10 min after the end of tDCS (*P* = 0.043, *d*_z_ = 0.83). We observed no other significant differences between the interventions during and after tDCS (*P*-values > 0.1).

#### The Effect of tDCS on the Right Finger

We conducted the same two-way ANOVA for the right finger trials and found a significant main effect of Intervention (*F*_(2,18)_ = 7.44, *p* = 0.004, $\eta_{\text{p}}^{2}$ = 0.45) and a significant interaction (*F*_(4,36)_ = 9.61, *P* < 0.001, $\eta_{\text{p}}^{2}$ = 0.52). *Post hoc* pairwise comparisons with Dunnett’s correction showed no significant difference between the interventions before tDCS was applied (*P*-values > 0.1) and after the end of tDCS (*P*-values > 0.2). Conversely, we found a significantly lower grating threshold in the Dual-Anode-Left condition compared with the sham condition during tDCS (*P* = 0.002, *d*_z_ = 2.34), and a significantly higher threshold in the Dual-Anode-Right condition compared with the sham condition during the same time period (*P* = 0.036, *d*_z_ = 0.69). No other significant differences were observed.

### The Effects of Dual-Hemisphere vs. Uni-Hemisphere tDCS

To test our main hypothesis, we then compared the effects of dual-hemisphere vs. uni-hemisphere tDCS. Figure 2 shows the threshold patterns. Two-way ANOVA (three levels of Intervention *×* three levels of Time) showed a significant interaction (*F*_(4,36)_ = 5.91, *p* = 0.001, $\eta_{\text{p}}^{2}$ = 0.40), as well as significant main effects of Intervention (*F*_(2,18)_ = 4.33, *p* = 0.029, $\eta_{\text{p}}^{2}$ = 0.32) and Time (*F*_(2,18)_ = 11.01, *P* = 0.001, $\eta_{\text{p}}^{2}$ = 0.55). We conducted *post hoc* pairwise comparisons with Dunnett’s correction (two-tailed) to compare the dual-hemisphere tDCS and sham with uni-hemisphere tDCS condition. This analysis revealed no significant differences between the interventions before tDCS was applied (*P* values > 0.8) and after the end of tDCS (*P*-values > 0.3). Conversely, we found a significantly lower grating threshold in the Dual-Anode-Left condition compared with the Uni-Anode-Left condition during tDCS (*P* = 0.017, *d*_z_ = 0.93). The comparison between the Uni-Anode-Left and Sham conditions showed a trend towards a significant difference (*P* = 0.054, *d*_z_ = 0.61). No other significant differences were observed.

### Questionnaire after Each Session

None of the participants reported side effects. Table 1 shows ratings of attention, fatigue, pain and discomfort reported by participants at the end of each intervention. A one-way ANOVA (four levels of Intervention) for each of the post-experimental ratings showed no significant differences (*P*-values > 0.1).

**Table 1:** Questionnaire scores after each intervention.

	Dual-Anodal-Left	Dual-Anodal-Right	Uni-Anodal-Left	Sham
Attention	1.3 ± 0.2	1.3 ± 0.2	1.1 ± 0.1	1.4 ± 0.2
Fatigue	1.2 ± 0.1	1.3 ± 0.2	1.2 ± 0.1	1.3 ± 0.2
Pain	1.1 ± 0.1	1.1 ± 0.1	1.1 ± 0.1	1.5 ± 0.2
Discomfort	1.3 ± 0.2	1.3 ± 0.2	1.3 ± 0.2	1.4 ± 0.2

*Data represent the group mean ± SEM. Attention was scored on a scale of 1–4 (1 = no distraction; 4 = highest level of distraction). Fatigue was scored on a scale of 1–4 (1 = no fatigue; 4 = highest level of fatigue). Pain was scored on a scale of 1–4 (1 = no pain; 4 = strongest pain). Discomfort was scored on a scale of 1–4 (1 = no discomfort; 4 = strongest discomfort). A one-way ANOVA (four levels of Intervention) for each of the post-experimental ratings showed no significant differences (P-values > 0.1)*.

### Electric Field Modeling

EF modeling revealed that the highest group-average EFs were located in regions in and around the lateral sulcus (Figures 3B,C). EFs in the PO and S1 hand area are shown in Figure 3D. As expected, EFs in the bilateral PO were symmetric in dual-hemisphere tDCS (*P*-values > 0.9), whereas the left PO showed stronger EFs than the right PO in uni-hemisphere tDCS (*t*_(61)_ = 17.56, *P* < 0.001, *d*_z_ = 2.23 for peak EF; *t*_(61)_ = 38.22, *P* < 0.001, *d*_z_ = 4.85 for mean EF).

We compared EFs in the PO with EFs in the S1 hand area. Both the peak and mean EFs were significantly higher in the left PO than in the hand area of the left S1 in dual-hemisphere tDCS (*t*_(61)_ = 11.42, *P* < 0.001, *d*_z_ = 1.45 for peak EF; *t*_(61)_ = 17.63, *P* < 0.001, *d*_z_ = 2.24 for mean EF) and in uni-hemisphere tDCS (*t*_(61)_ = 9.24, *P* < 0.001, *d*_z_ = 1.17 for peak EF; *t*_(61)_ = 22.08, *P* < 0.001, *d*_z_ = 2.80 for mean EF).

## Discussion

In the present study, we examined the effects of dual-hemisphere tDCS over the PO on tactile discrimination of grating orientation. The mean grating orientation threshold without tDCS ranged between 0.9 mm and 1.1 mm, similar to the values reported in an earlier grating orientation discrimination study (Van Boven and Johnson, 1994), but slightly lower than the thresholds reported in some previous studies (around 1.2 mm; Vega-Bermudez and Johnson, 2001; Fujimoto et al., 2014, 2016). This difference may be partially due to practice effects; the participants in the present study completed four sessions with different interventions, potentially resulting in better performance in later sessions. However, because we counterbalanced the order of the sessions across participants, such learning effects are expected to be equally reflected in all conditions. Therefore, it is unlikely that the effects of dual-hemisphere tDCS, shown as the difference from other interventions, were due to such practice effects.

As expected, we found that dual-hemisphere tDCS over the PO elicited a lower tactile discrimination threshold compared with sham stimulation. This is consistent with our previous finding that dual-hemisphere tDCS over the PO in stroke patients decreased the tactile discrimination threshold for affected fingers (Fujimoto et al., 2016). Compared with this previous study, the present study produced two new findings. First, in the present study, we demonstrated an improved orientation threshold for both fingers; tDCS with left and right anode electrodes decreased the threshold of the right and left fingers, respectively. Second, tDCS with the anode electrode over the right PO increased the threshold of the right finger during tDCS. These results indicate that dual-hemisphere tDCS over the PO can increase or decrease the tactile discrimination of grating orientation in both the left and right hands.

Previous studies have shown that tDCS over the somatosensory cortices can affect SEPs (Matsunaga et al., 2004; Dieckhofer et al., 2006; Sugawara et al., 2015; Lenoir et al., 2017; Nakagawa et al., 2017), tactile discrimination of vibratory stimuli (Rogalewski et al., 2004) and spatial acuity (Ragert et al., 2008; Fujimoto et al., 2014, 2016; Hilgenstock et al., 2016). Taken together with previous findings, the current results support the efficacy of tDCS for affecting somatosensory function.

The main purpose of the present study was to compare the effects of dual-hemisphere tDCS with those of uni-hemisphere tDCS. We found that the effect of dual-hemisphere tDCS on grating orientation discrimination was stronger than the effect of uni-hemisphere tDCS. This indicates that inhibition of the ipsilateral PO enhances tactile orientation perception in the hand, supporting the hypothesis that IHI occurs in the PO. We previously showed that dual-hemisphere tDCS over S1 improved the grating threshold compared with uni-hemisphere tDCS (Fujimoto et al., 2014). In the current study, we extended this finding by showing that the advantage of dual-hemisphere tDCS can be applied not only to S1, but also to the PO, which contains S2.

Compared with S1, the PO more frequently exhibits bilateral activation in neuroimaging studies when only one hand (right hand) is stimulated (Karhu and Tesche, 1999; Bodegård et al., 2000; Burton et al., 2004; Kitada et al., 2005; Yang et al., 2017). The SEPs of the ipsilateral PO are reported to be 13 ms slower than the contralateral PO (Karhu and Tesche, 1999). One straightforward explanation for this finding is that the contralateral PO activates the ipsilateral PO via the corpus callosum. However, IHI indicates that activity in the contralateral PO should decrease, not increase, activity in the ipsilateral PO. Alternatively, the ipsilateral PO may receive signals from other brain regions. In accord with this notion, the PO in non-human primates is anatomically connected to a number of different regions, including the posterior parietal lobule (Disbrow et al., 2003). The posterior parietal lobule shows bilateral activation during orientation discrimination (Kitada et al., 2006, 2014). Thus, ipsilateral PO may receive unwanted activity from the posterior parietal lobule and respond by inhibiting activity in the contralateral PO. Dual-hemisphere tDCS may inhibit such activity in the ipsilateral PO, which in turn enhances activity in the contralateral PO. This additive enhancement may produce a difference in task performance between dual-hemisphere tDCS and uni-hemisphere tDCS.

In the present study, we adopted the GOT to examine the effect of tDCS over the PO. Our result is consistent with previous findings in non-human primates (Fitzgerald et al., 2006; Thakur et al., 2006). These studies reported that neurons in the S2 of non-human primates are tuned to the specific orientation of an object contacted by the hand. Thus, the contralateral PO may play an important role in processing orientation. However, several neuroimaging studies have demonstrated that activity in the PO is associated with the perception of material properties of an object (Craig et al., 2000; Kitada et al., 2005; Eck et al., 2016). Further, this region is reported to be more strongly activated by material properties compared with macrospatial properties, such as shape (Roland et al., 1998; Stilla and Sathian, 2008). The PO is also known to play a role in pain processing, although we found no effect in our previous study (Koyama et al., 2017). Therefore, future studies should directly compare the effects of tDCS on perception of object properties and pain.

### The Focality of Stimulation

EF modeling indicated that both dual-hemisphere and uni-hemisphere tDCS stimulated the region in and around the PO. In S1, for instance, the face area is the region most adjacent to the PO, and thus could be affected by tDCS. However, the S1 hand area is approximately 3 cm away from the PO and the effects of stimulation over this area was weaker than for the PO. Indeed, the same tDCS protocol used in the present study has been found to affect SEPs in the PO but not in the S1 (Nakagawa et al., 2017). Thus, although we cannot rule out the contribution of the S1 hand area, its role is minor compared to the PO. Similarly, the inferior parietal lobule is located postero-superior to the PO. This region is involved in orientation discrimination and shows right hemisphere laterality (Kitada et al., 2006). If the effect of the tDCS is mainly from activity in this region, such laterality would be expected to be reflected in task performance. However, such an effect was not clearly observed, suggesting that the influence of tDCS on the inferior parietal lobule is smaller than that on the PO. Nevertheless, it is necessary to test the relative contributions of these adjacent areas in future work.

### The Sample Size

One of the potential issues is the small number of the participants (10 participants). This number was determined according to our previous studies that demonstrated the effect of the dual-hemisphere tDCS over the somatosensory cortices on tactile orientation discrimination in stroke patients (Fujimoto et al., 2016) and healthy participants (Fujimoto et al., 2014). Indeed, although these previous studies involved similar sample sizes, they successfully demonstrated the effect of dual-hemisphere tDCS on their behavioral performance. As we used double-blinded design, the effect in the present study cannot be explained by any observer effect. More importantly, we found the tDCS effect in the majority of the individual data (Figures 1C, 2B), as well as the group result. Thus, although it is important to replicate our findings in the future, it is unlikely that our result is biased by a small number of the participants.

### Limitations and Future Consideration

Several important limitations should be considered when interpreting the current results. First, task performance was not significantly different in the left finger between sham and dual-hemisphere tDCS (with the anode on the left scalp). Thus, future work should investigate the possible asymmetric effects of dual-hemisphere tDCS. Second, we only compared the effects of dual-hemisphere tDCS with those of uni-hemisphere tDCS on the right finger, because of hand laterality. Indeed, most previous neuroimaging studies examining ipsilateral PO activation stimulated only the right hand (Roland et al., 1998; Karhu and Tesche, 1999; Kitada et al., 2005; Stilla and Sathian, 2008; Yang et al., 2017). However, confirming this result in future studies by testing the effects of uni-hemisphere tDCS on the left finger will be critical for clarifying this issue. Finally, the numbers of male and female participants in the experiment were not matched. Given the previously reported sex differences in tactile spatial acuity (Goldreich and Kanics, 2003), it may be useful to examine sex differences in the effects of tDCS over the PO.

Despite these shortcomings, the current findings may contribute to the development of therapeutic approaches for recovering somatosensory function following somatosensory cortex damage. Indeed, we demonstrated that the effects of tDCS can last for 10 min after the end of the stimulation, in accord with previous findings (Fujimoto et al., 2016). Thus, the results of the present study constitute an important step toward the clinical application of tDCS over the PO. In conclusion, we compared the effects of dual-hemisphere tDCS with those of conventional uni-hemisphere tDCS over the PO. We observed a greater improvement in the threshold for tactile grating orientation discrimination with dual-hemisphere vs. uni-hemisphere tDCS. This result indicates that inhibition of the ipsilateral PO enhances tactile orientation perception, supporting the hypothesis that the bilateral PO may function in competition, rather than in harmony.

## Author Contributions

ST and RK conceived and supervised the study. SF, ST and RK designed the experiments. SF, TY, NK and KK carried out the experiments. SF, NK, RK, IL and ST analyzed the data. SF, RK, IL, TN and ST wrote the manuscript. All authors approved the final version of the submitted manuscript.

## Conflict of Interest Statement

The authors declare that the research was conducted in the absence of any commercial or financial relationships that could be construed as a potential conflict of interest.
