# Supplementary material for: The Effect of Dual-Hemisphere Transcranial Direct Current Stimulation Over the Parietal Operculum on Tactile Orientation Discrimination
Source: Front Behav Neurosci. 2017 Sep 20;11:173. doi: 10.3389/fnbeh.2017.00173 (PMC5611440; doi:10.3389/fnbeh.2017.00173)
Supplement: Supplementary file 1 [file Table_1.doc]

**Supplemental Table 1 Individual data on the left finger**

|  | Dual-Anode-Left | | | Dual-Anode-Right | | | Sham | | |
| --- | --- | --- | --- | --- | --- | --- | --- | --- | --- |
| Participant | Pre | During | Post10min | Pre | During | Post10min | Pre | During | Post10min |
| 1 | 0.90 | 1.05 | 1.00 | 0.89 | 0.75 | 0.75 | 0.88 | 0.88 | 0.88 |
| 2 | 1.35 | 1.32 | 1.50 | 1.20 | 0.58 | 1.00 | 1.20 | 1.16 | 1.20 |
| 3 | 1.20 | 1.20 | 1.00 | 1.50 | 0.75 | 0.92 | 1.40 | 1.50 | 1.40 |
| 4 | 0.86 | 0.91 | 0.82 | 0.88 | 0.46 | 0.50 | 0.88 | 0.82 | 0.86 |
| 5 | 1.35 | 1.50 | 0.88 | 0.88 | 0.64 | 0.68 | 0.94 | 1.00 | 1.05 |
| 6 | 1.00 | 0.98 | 1.10 | 1.10 | 0.75 | 1.05 | 1.35 | 0.89 | 0.97 |
| 7 | 0.84 | 0.88 | 0.82 | 0.96 | 0.48 | 0.50 | 0.88 | 0.88 | 0.91 |
| 8 | 0.91 | 0.91 | 0.90 | 0.91 | 0.63 | 0.80 | 0.86 | 0.88 | 0.89 |
| 9 | 1.33 | 1.29 | 0.69 | 1.26 | 0.67 | 1.26 | 1.20 | 1.10 | 1.00 |
| 10 | 0.81 | 0.92 | 0.75 | 0.80 | 0.48 | 0.46 | 0.83 | 0.75 | 0.69 |
|  |  |  |  |  |  |  |  |  |  |
| Mean | 1.06 | 1.09 | 0.95 | 1.04 | 0.62 | 0.79 | 1.04 | 0.98 | 0.98 |
| SEM | 0.07 | 0.07 | 0.07 | 0.07 | 0.04 | 0.08 | 0.07 | 0.07 | 0.06 |
